# The journey of stage III and IV non-small cell lung cancer patients in the Brazilian private healthcare system: a retrospective study

**DOI:** 10.3389/fonc.2023.1257003

**Published:** 2023-10-18

**Authors:** Erica R. Cerqueira, Paula M. Batista, Milena F. Almeida, Maria A. C. Rego, Ana C. P. Ribeiro-Pereira, Fernando Alencar, Roberta A. Fernandes, Aknar F. C. Calabrich, Gustavo Schvartsman

**Affiliations:** ^1^ Global Medical Scientific Affairs, MSD Brazil, São Paulo, Brazil; ^2^ Department of Health Economics, Origin Health, São Paulo, Brazil; ^3^ Department of Thoracic Oncology, Clínica AMO, Salvador, Bahia, Brazil; ^4^ Department of Medical Oncology, Hospital Israelita Albert Einstein, São Paulo, Brazil

**Keywords:** Lung cancer1, Brazil2, RWE3, Access4, Treatment5

## Abstract

Non-small cell lung cancer (NSCLC) is still diagnosed at late stages in Brazil. The availability of newer treatment options has changed patient management, however, few real-world data have been published since then. This is a population-based retrospective cohort study that aims to evaluate the characteristics of stage III/IV NSCLC patients and their journey in the Brazilian private healthcare system. Patients aged ≥18 years, residing in Brazil who had their first medical appointment between 2016 and 2018 were included in the study. The sociodemographic and clinical characteristics of the patients and time intervals of interest were described. A total of 10,394 patients were analyzed. The majority of the patients were male (58.5%) with a median age of 64.0 (IQR = 58.0 – 71.0) years. In relation to characteristics of the disease, most of the tumors were characterized as adenocarcinomas (52.3%) and diagnosed at stage IV (72.2%). Most patients arrived at the hospital with an established NSCLC diagnosis, while 45.7% were diagnosed at the first medical appointment in the hospital or later. For patients who were diagnosed at the first medical appointment or later, a median interval of 15.0 (IQR = 6.0 – 33.0) days was observed between the first medical appointment and the diagnosis. The first treatment was given after a median of 25.0 (IQR = 6.0 – 49.0) days after diagnosis for patients without a prior diagnosis, and 57.0 (IQR: 33.0 – 98.0) days for patients with a prior diagnosis. The most common treatments were chemotherapy alone (33.8%), chemotherapy combined with radiotherapy (21.5%), radiotherapy alone (13.1%), adjuvant or neoadjuvant treatment (9.3%), surgery (3.3%), and immunotherapy (0.7%; alone or combined). At the end of follow-up (September, 2020), 52.3% of the patients had died. Despite having more treatment options in the private sector, data show that there is a need to improve access to technologies.

## 1 Introduction

Lung cancer is the leading cause of cancer death globally, with an estimated 1.8 million deaths (18%) in 2020 (1). In Brazil, lung cancer may be the second or third most common cancer among men, depending on the country region, and the third most common cancer among women. As in most countries, lung cancer is the leading cause of cancer mortality in Brazil (2). NSCLC is usually diagnosed in advanced stages and has poor survival rates in Brazil. A study has shown that roughly 87% of the patients were diagnosed with locally advanced or metastatic disease (stages III and IV, respectively) between 2000 and 2014 (3, 4).

Considering that NSCLC is still diagnosed at later stages, some studies have been conducted in order to understand the patients’ journey (5–7). Data from Greece and Bangladesh report a prolonged interval from the onset of symptoms to treatment; within 81 and 151 days, respectively (5, 7). To deal with this issue in Canada, Cotton et al. (2020) proposed a diagnostic assessment program to improve the efficiency of the healthcare system and reported a decrease in the wait time to diagnosis (8).

There is a lack of reliable structured and centralized data on lung cancer treatment in Brazil, with very high rates of misdiagnosis and underreporting throughout the country (9). The Brazilian healthcare system is divided into two main players, the public sector represented by the Unified Healthcare System, and the private sector, represented for the most part by the Supplementary Healthcare System (10). Different reimbursement processes are defined for sectors and, the private sector has greater access to high complexity and innovative drugs. The purpose of the present study is to provide information on the journey of stage III/IV NSCLC patients and their characteristics in the Brazilian private healthcare system between 2016 and 2018.

## 2 Materials and methods

This is a population-based retrospective cohort study to characterize the journey of stage III/IV NSCLC patients in the Brazilian private healthcare system. For the present study, adult patients aged ≥18 years, residing in Brazil, diagnosed with stage III/IV NSCLC, as reported in the database, whose hospital data was sent to the Cancer Hospital Registry (from Portuguese, *Registro Hospitalar de Câncer* – RHC) database, who had their first medical appointment between January 2016 and December 2018 were included and followed until Sept./17/2020. Exclusion criteria were defined as follows: absence of “C34” (lung cancer code); absence of histological type compatible with NSCLC; and patients treated exclusively in the public sector. The database was checked for inconsistencies and the following data were excluded: individuals diagnosed ≥1 year before the first medical appointment, those treated with hormone therapy, and those who received transplants.

The databases considered for the retrospective study were extracted using the ICD-10 code C34 (C34.0, C34.1, C34.2, C34.3, C34.8 and C34.9) for lung cancer and histology type to identify NSCLC (e.g., adenocarcinoma and squamous cell carcinoma). This study focused on the private sector of the Brazilian healthcare system. RHC is a hospital-based cancer registry that systematically and continuously collects, stores, processes and analyzes information from patients treated in a hospital unit with a confirmed diagnosis of cancer (11).

The study was conducted in accordance with the requirements of CNS/MS Resolution No. 466/2012 (12). The data were obtained from a public secondary database, therefore approval by an ethics committee was waived. The assessment and treatment of the cases are based on a private database, which is anonymized, protecting the confidentiality and privacy of the patients. Considering the private context of the data, a formal Consent to Release Information is not required as per Resolution 510/2016 Art.14 (12).

The date of the first medical appointment is defined as the date of patient care by the service responsible for their diagnosis/treatment, as well as the first contact with the doctor who initiates the diagnostic/therapeutic process. Visits for screening are not included. The first diagnosis date is defined as the date of anatomopathological confirmation of the tumor. When the patient arrives at the hospital with an established diagnosis, all information sent through the medical report, or tests performed at other institutions or other services must be used. The year of the first medical appointment defines the year of the database in which patients will be registered (11).

For data analysis, the data was initially checked to exclude cases with inconsistences such as patients diagnosed one year or more before the first medical appointment, patients treated with hormone therapy and transplant patients. The dates informed in the database were used to estimate specific variables as follows: “interval between the first medical appointment and diagnosis” (using “date of the first medical appointment” and “date of the first diagnosis”), “interval between the diagnosis and first treatment” (“date of the first diagnosis” and “date of the start of the first specific treatment for the tumor at the hospital”) and “interval between the first treatment and death” (“date of the start of the first specific treatment at the hospital” and “date of death”).

A convenience sample was considered for this study and all patients with inclusion criteria were filtered and included. An initial descriptive statistical analysis was performed, and continuous variables were checked for normality distribution in order to select the most appropriate analysis technique. Qualitative or categorical variables were summarized using descriptive statistics using the number of observations (n) and percentages (%). The Mann-Whitney test was used to verify the association of tumor histological type and tumor stage with the interval between the first medical appointment and diagnosis, interval between the diagnosis and the first treatment, and interval between the first treatment and death.

## 3 Results

**Figure 1:**
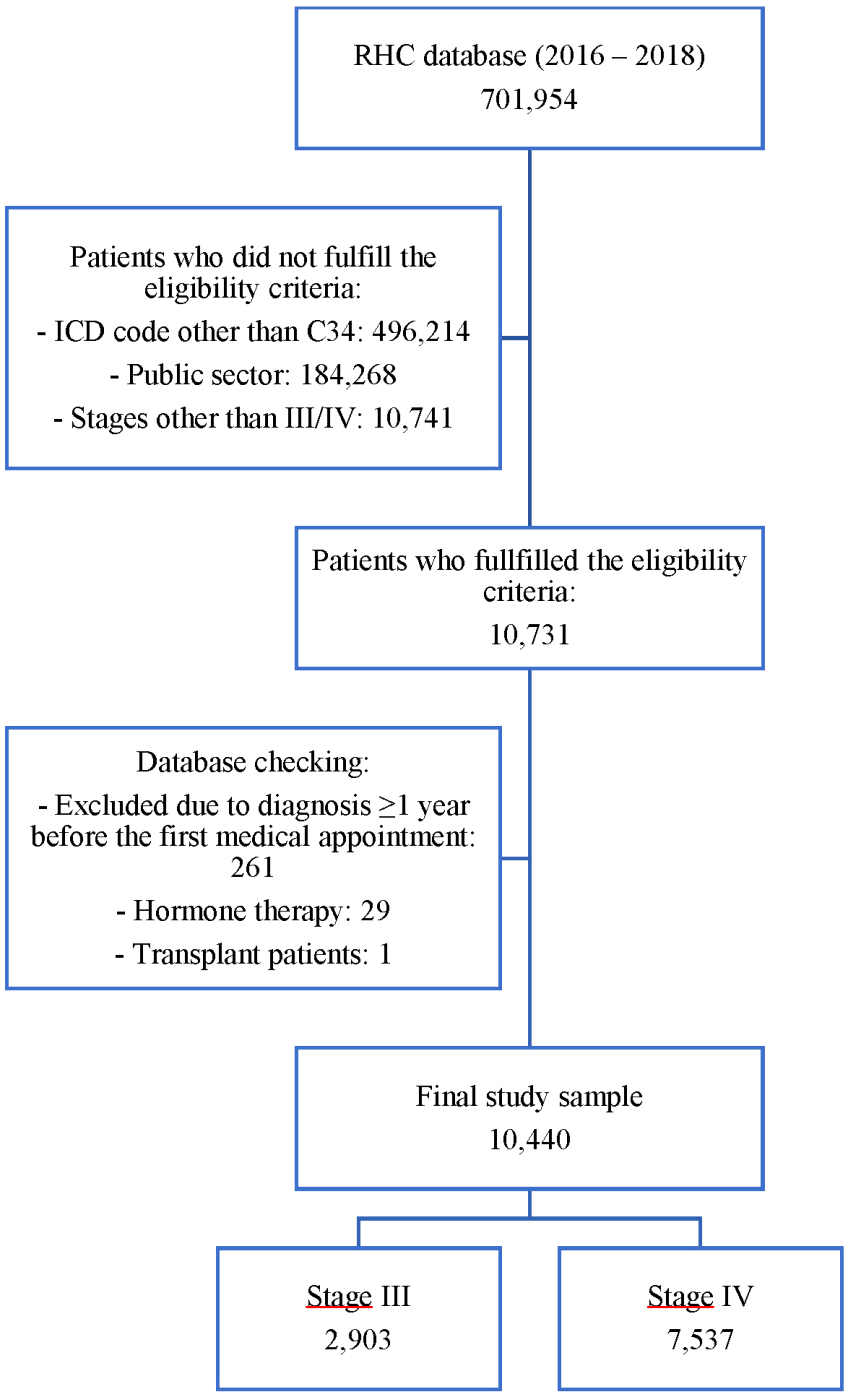
Flowchart of the number of records in the database after fulfilling the inclusion and exclusion criteria.

The RHC database retrieved a total of 701,954 records between 2016 and 2018. After applying the eligibility criteria, 10,731 registries were analyzed. The database was checked to exclude inconsistent data. A total of 10,440 patients were included in the study. **Figure 1** shows the study sample flowchart, including reasons for exclusion.

**Table 1** shows general characteristics of the study sample. The median age was 64.0 years (interquartile range [IQR]: 58.0 – 71.0), and most individuals were male (58.5%), caucasian (34.7%), with incomplete junior high school education (41.5%) and residing in the Southeastern Region (51.9%). In regard to patient acceptance, 24.5% came from an oncology clinic.

**Table 1:** Sociodemographics of stage III/IV NSCLC patients.

	n	%
Age at the first medical appointment		
**Median/IQR**	64.0 years	58.0 - 71.0 (years)
Sex		
**Female**	4,331	41,5
**Male**	6,109	58,5
Education		
**None**	810	7,8
**Incomplete junior high school education**	4,335	41,5
**Junior high school education**	1,752	16,8
**High school**	1,435	13,7
**Undergraduate student**	41	0,4
**Graduate**	592	5,7
**No information**	1,475	14,1
Ethnicity		
**Caucasian**	3,618	34,7
**Hispanic**	2,527	24,2
**Black**	307	2,9
**Asian**	53	0,5
**Native**	5	0,0
**No information**	3,930	37,6
Region where the patient resides		
**Southeast**	5,422	51,9
**South**	2,848	27,3
**Northeast**	1,615	15,5
**Midwest**	450	4,3
**North**	66	0,6
**No information**	39	0,4
First treatment clinic (patient acceptance)		
**Oncology clinic**	2,555	24,5
**Thoracic surgery**	1,172	11,2
**Radiotherapy**	923	8,8
**Pneumology**	469	4,5
**Surgical oncology**	396	3,8
**Medical clinic**	281	2,7
**Screening**	242	2,3
**Other**	352	3,4
**No information**	4,050	38,8

IQR, Interquartile range.

**Table 2** presents information on the tumor, treatment and other clinical characteristics. Adenocarcinoma was the most common histological tumor type (52.3%), followed by others (43.8%) and squamous cell carcinomas (3.9%). For 72.2% of the patients, staging at diagnosis was IV. The most significant basis for tumor diagnosis was primary tumor histology in 57.7% of the cases, followed by histology of metastasis (4.7%) and image for examination (1.4%). In addition, family history was reported by 2,010 patients (19.3%), corresponding to more than half of the patients who reported a family history.

**Table 2:** Tumor, treatment and other clinical data of stage III/IV NSCLC patients.

	n	%
Tumor histological type		
**Adenocarcinoma**	5,465	52,3
**Squamous cell carcinoma**	402	3,9
Other*	4,573	43,8
Staging at the first assessment		
**3**	2,903	27,8
**3 – undefined subgroup**	35	0,3
**3A**	1,616	15,5
**3B**	1,250	12,0
**3C**	2	0,0
**4**	7,537	72,2
**4 - undefined subgroup**	7,511	71,9
**4A**	9	0,1
**4B**	12	0,1
**4C**	5	0,0
Most significant basis for tumor diagnosis		
**Histology of the primary tumor**	6,029	57,7
**Histology of metastasis**	486	4,7
**Image for examination**	144	1,4
**Cytology**	70	0,7
**Clinical research**	23	0,2
**Clinic**	13	0,1
**Tumor markers**	10	0,1
**No information**	3,665	35,1
Family history of cancer		
**Yes**	2,010	19,3
**No**	1,809	17,3
**No information**	6,621	63,4

*Composite carcinoid tumor; carcinoma, anaplastic type NOS; giant cell carcinoma; polygonal cell carcinoma; carcinoma, undifferentiated NOS; pleomorphic carcinoma; large cell carcinoma NOS; carcinoma NOS; large cell carcinoma, rhabdoid; non-small cell carcinoma; solid carcinoma NOS; carcinoma, metaplastic NOS.

**Table 3** presents the results related to primary outcomes of the study. Most patients (53.7%) were diagnosed before their first medical appointment. For patients diagnosed at their first medical appointment or later (45.7%), a median interval of 15 days (IQR: 6 – 33) was observed between the first medical appointment and diagnosis. The first treatment was given after a median interval of 25 days (IQR: 6 – 49) after diagnosis for patients without a prior diagnosis, and 57 days (IQR: 33 – 98) for patients with a prior diagnosis. Most patients were treated with chemotherapy alone (33.5%), chemotherapy combined with radiotherapy (21.5%) and radiotherapy alone (13.4%). In addition, 9.3% of patients were treated with neoadjuvant or adjuvant treatments, 3.3% with surgery, and 0.7% with immunotherapy (alone or combined), while 16.3% did not receive any therapeutic strategy. A complete description of the first treatment strategy used is shown in the **Supplementary Table 1**. A median interval of 124 (IQR: 41 – 275) days was seen between the first treatment and death. Data was stratified according to stage at diagnosis and is also shown in **Table 3**.

**Table 3:** Amount of time before diagnosis, treatment and death, of stage III/IV NSCLC patients.

	Overall	Stage III	Stage IV
	N (%)	N (%)	N (%)
Interval between the first medical appointment and diagnosis (months)*			
**Diagnosis at the first medical appointment or later**	4,765 (45.6)	1,301 (44.8)	3,464 (46.0)
**≤30 days**	3,473 (33.3)	860 (29.6)	2,613 (34.7)
**>30 days and ≤90 days**	1,024 (9.8)	354 (12.2)	670 (8.9)
**>90 days and ≤180 days**	205 (2.0)	66 (2.3)	139 (1.8)
**>180 days and ≤360 days**	47 (0.5)	15 (0.5)	32 (0.4)
**<720 days**	15 (0.1)	05 (0.2)	10 (0.1)
**≥720 days**	01 (0.0)	01 (0.0)	00 (0.0)
**Median/IQR**	15 (6.0 - 33.0)	18 (7.0 - 41.0)	14 (6.0 - 30.0)
**Diagnosis prior to the first consultation**	5,605 (53.7)	1,586 (54.6)	4,019 (53.3)
**No information**	70 (0.7)	16 (0.6)	54 (0.7)
Interval between the diagnosis and first treatment (months)*			
**Diagnosis at the first medical appointment or later**	3,846 (36.8)	1,105 (38.1)	2,741 (36.4)
**≤30 days**	2,228 (57.9)	537 (48.6)	1,691 (61.7)
**>30 days and ≤90 days**	1,289 (33.5)	438 (39.6)	851 (31.0)
**>90 days and ≤180 days**	270 (7.0)	111 (10.0)	159 (5.8)
**>180 days and ≤360 days**	50 (1.3)	16 (1.4)	34 (1.2)
**<720 days**	09 (0.2)	03 (0.3)	06 (0.2)
**Median/IQR**	25 (6.0 - 49.0)	33 (10.0 - 60.0)	22 (5.0 - 45.0)
**Diagnosis prior to the first consultation**	4,661 (44.6)	1,404 (48.4)	3,257 (43.2)
**≤30 days**	1,053 (22.6)	273 (19.4)	780 (23.9)
**>30 days and ≤90 days**	2,289 (49.1)	665 (47.4)	1,624 (49.9)
**>90 days and ≤180 days**	918 (19.7)	337 (24.0)	581 (17.8)
**>180 days and ≤360 days**	353 (7.6)	109 (7.8)	244 (7.5)
**<720 days**	48 (1.0)	20 (1.4)	28 (0.9)
**Median/IQR**	57 (33.0 - 98.0)	64 (36.0 - 110.0)	54 (32.0 - 93.0)
**No information/Not applicable**	1,933 (18.5)	394 (13.6)	1,539 (20.4)
First therapeutic strategy received at the hospital			
**Chemotherapy only**	3,528 (33.8)	837 (28.8)	2,691 (35.7)
**Chemotherapy combined with radiotherapy (concomitant or sequential)**	2,242 (21.5)	897 (30.9)	1,345 (17.8)
**Radiotherapy only**	1,371 (13.1)	390 (13.4)	981 (13.0)
**Adjuvant or neoadjuvant treatment**	972 (9.3)	287 (9.9)	685 (9.1)
**Surgery**	342 (3.3)	94 (3.2)	248 (3.3)
**Immunotherapy alone or in combination with other modalities**	73 (0.7)	14 (0.5)	59 (0.8)
**Other**	194 (1.9)	43 (1.5)	151 (2.0)
**None**	1,706 (16.3)	339 (11.7)	1,367 (18.1)
**No information**	12 (0.1)	02 (0.1)	10 (0.1)
Interval between first treatment and death (months)*			
**≤30 days**	875 (8.4)	150 (5.2)	725 (9.6)
**>30 days and ≤90 days**	958 (9.2)	176 (6.1)	782 (7.5)
**>90 days and ≤180 days**	849 (8.1)	188 (6.5)	661 (6.3)
**>180 days and ≤360 days**	999 (9.6)	242 (8.3)	757 (7.3)
**<720 days**	578 (5.5)	168 (5.8)	410 (3.9)
**≥720 days**	134 (1.3)	48 (1.7)	86 (0.8)
**Median/IQR**	124 (41.0 - 275.0)	166,5 (60.0 - 337.3)	112 (38.0 - 259)
**No information¹**	6,047 (57.9)	1,931 (66.5)	4,116 (54.6)

*The number of months was defined as the number of days divided by 30.

**Figure 2:**
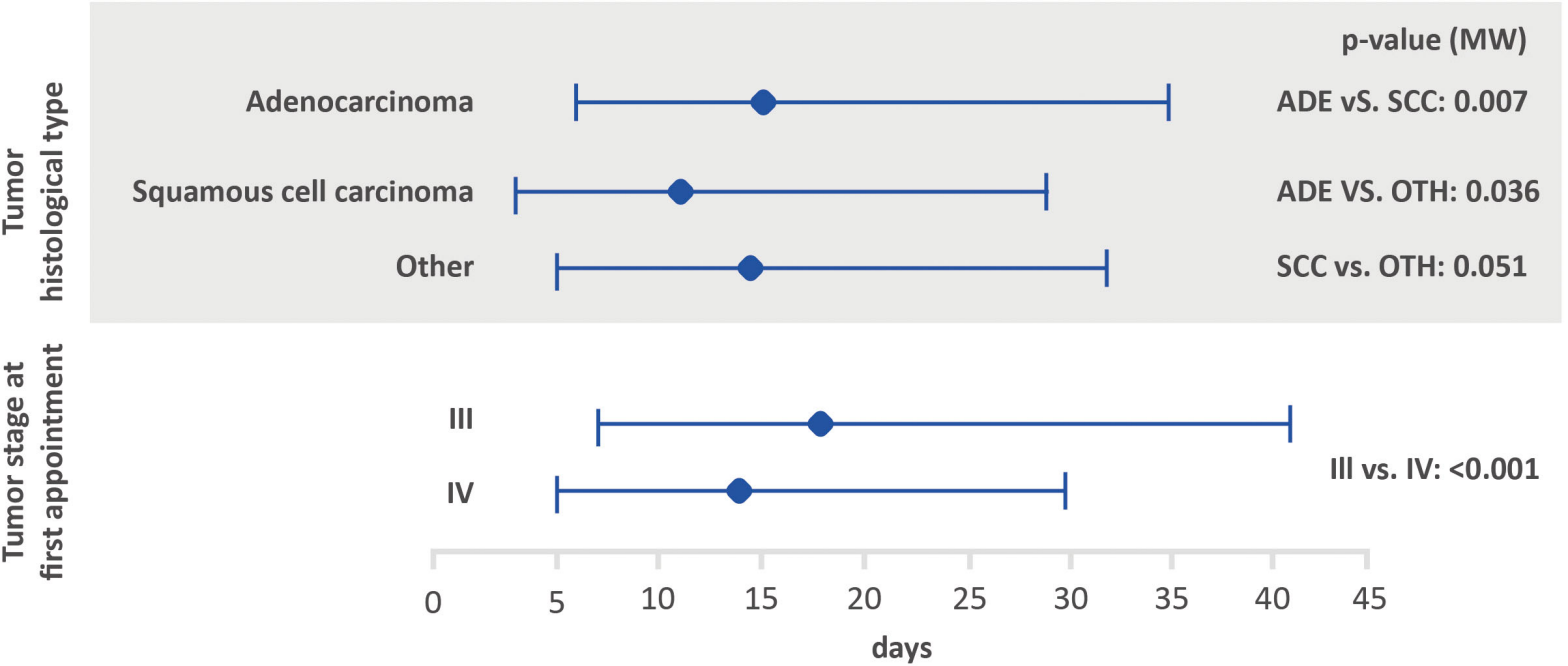
Amount of time between the first medical appointment and diagnosis, based on tumor histological type and stage at the first appointment using the Mann-Whitney test (lower bar limit: P25%; upper bar limit: P75%; square: median). ADE, adenocarcinoma; SCC, squamous cell carcinoma.

**Figure 3:**
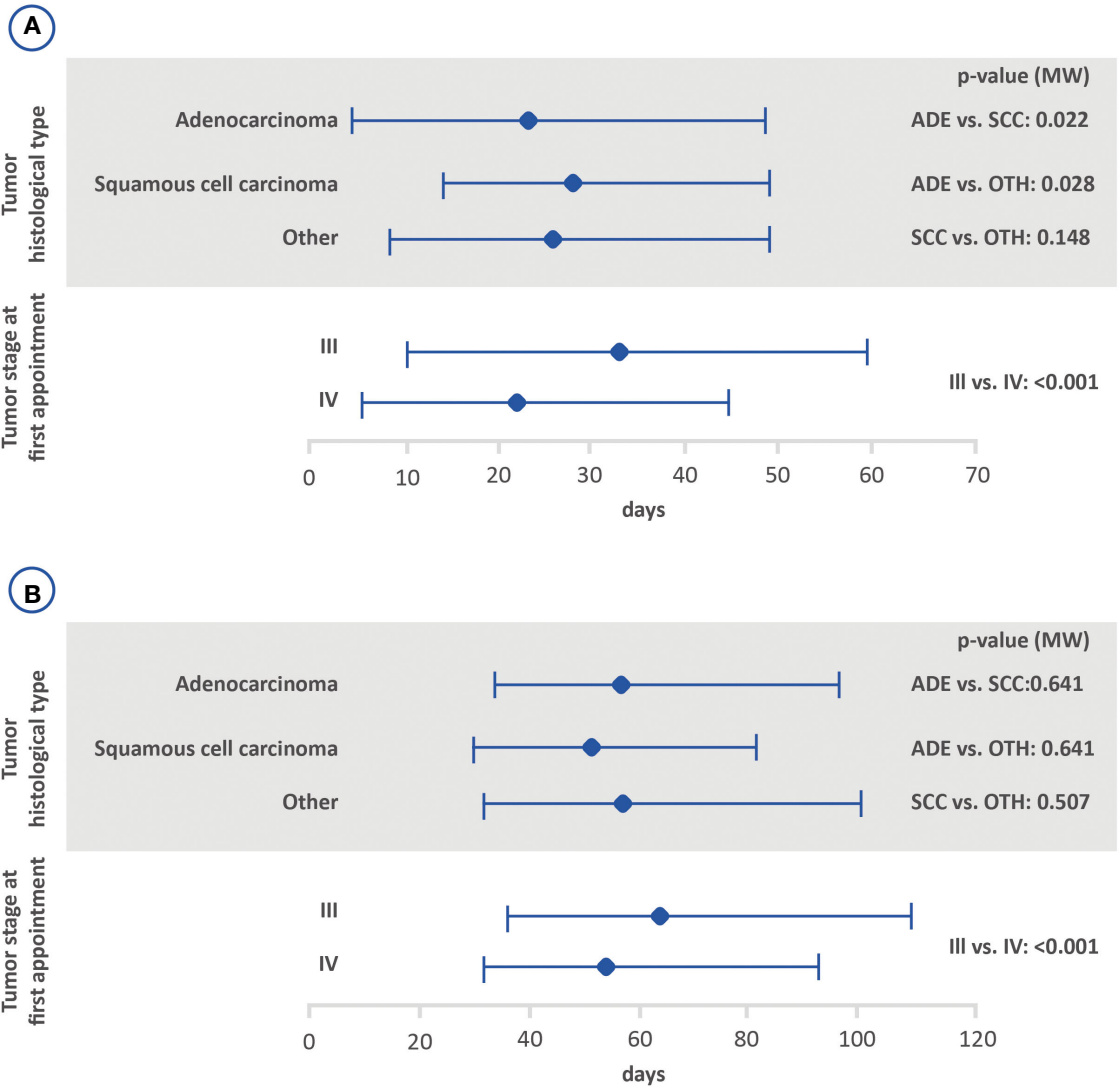
Amount of time between the diagnosis and the first treatment with **(B)** or without **(A)** prior diagnosis based on the histological tumor type and stage at the first appointment using the Mann-Whitney test (lower bar limit: P25%; upper bar limit: P75%; square: median). ADE, adenocarcinoma; SCC, squamous cell carcinoma.

**Figure 4:**
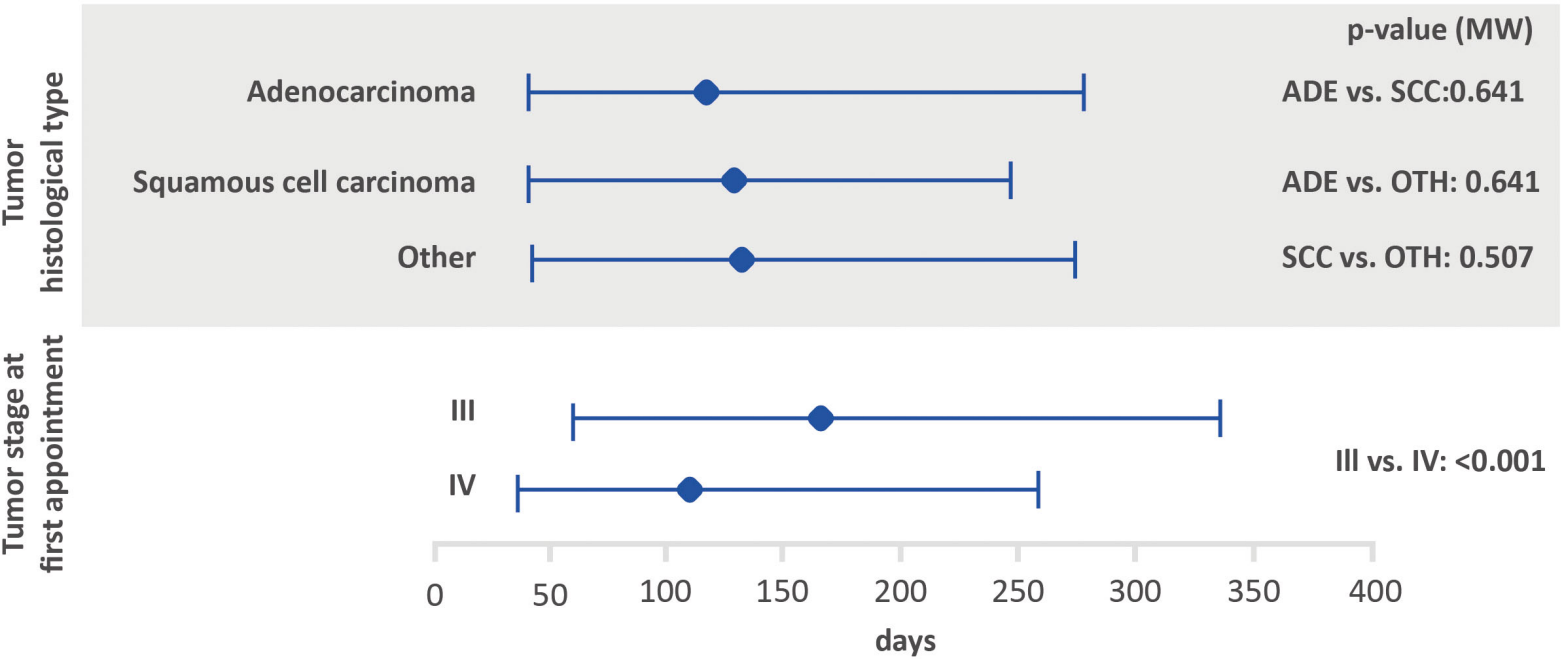
Amount of time between the first treatment and death based on histological tumor type and stage at the first appointment using the Mann-Whitney test (lower bar limit: P25%; upper bar limit: P75%; square: median). ADE, adenocarcinoma; SCC, squamous cell carcinoma.

Possible associations between the interval between the first medical appointment and diagnosis, interval between diagnosis and the first treatment and interval between the first treatment and death, with tumor stage and histological tumor type are shown in **Figures 2**–**4**. The interval between the first medical appointment and diagnosis, and interval between diagnosis and the first treatment (with diagnosis at the first medical appointment or later) observed for the adenocarcinoma histological type (median of 15 and 23 days, respectively) was significantly different in relation to squamous cell carcinomas (median of 11 and 28 days, respectively) and others (median of 14 and 26 days, respectively).

Regarding tumor stage at first appointment, a statistically significant difference was observed for all outcomes. The estimated values were 18 and 14 days for the interval between the first medical appointment and diagnosis for stage III and IV, respectively, 33 and 22 days for the interval between diagnosis and the first treatment (with diagnosis at the first medical appointment or later), 64 and 54 days for the interval between diagnosis and the first treatment (with the diagnosis prior to the first medical appointment) and 166 and 112 days for the interval between the first treatment and death.

## 4 Discussion

The purpose of this study was to evaluate the characteristics of stage III/IV NSCLC patients and their journey in the Brazilian private healthcare system. Overall survival after the first treatment was also evaluated. This analysis is essential to understand improvements in the management of NSCLC in the Brazilian private healthcare setting.

The Brazilian healthcare system is composed of two main players, the public health assistance provided by the Unified Healthcare System (*Sistema Único de Saúde* - SUS) and the private health assistance provided by the Brazilian private healthcare system (10). Roughly 47 million Brazilians use the private system, which comprises a mere 25% of the country’s population (13). Considering that different reimbursement and treatment access patterns are observed, the assessment of NSCLC patients based on the healthcare system used, provides robust information.

We analyzed 10,440 NSCLC patients stage III and IV. This number is close to the one published by GLOBOCAN 2020 that shows a prevalence of 43,726 lung cancer patients in 5 years. As we consider just the private care system (25% of the population) (13), we expect 10,931 lung cancer patients. However, our study was focused on NSCLC that represents 80% of all lung cancer (14), it means 8,745 patients according to GLOBOCAN data. Our number is a little higher than that meaning a good estimation of the NSCLC population in Brazil.

In relation to the patients’ journey up to the diagnosis of the disease, roughly 45% of overall, stage III and stage IV patients were diagnosed after their first medical appointment, and there was a median interval of 15 days between the events for overall and stage IV patients and 18 days for stage III. However, limitations inherent to the database must be considered when interpreting the results. Ferreira et al. (2019) reported on the journey of NSCLC patients in the private system between 2011 and 2016, finding that most of patients were diagnosed at stage IV, and the average interval between admission and diagnosis was 31 days (15). Abrao et al. (2017) published an analysis on the access of lung cancer patients to the Brazilian public healthcare system by reviewing the medical records of individuals followed between July/2008 and December/2014 in São Paulo. The estimated time between the onset of symptoms and diagnosis was roughly three months (16). It was evident that the interval between the medical appointment and diagnosis in the private system was much shorter, regardless disease stage.

Ferreira et al. (2021) have previously conducted a study to describe the journey of NSCLC patients in the Brazilian private healthcare system. However, the availability of newer treatment options, such as tyrosine kinase inhibitors (TKIs) and immunotherapy, could have had an impact on patient management in the country (6).

In this study, the median time to treatment (from diagnosis to start of treatment) for patients with stage III and IV NSCLC was 25 days for those without a prior diagnosis and 57 days for those with a prior diagnosis. The median time to treatment found for individuals without a prior diagnosis in this study concurs with prior studies that analyzed the time to treatment for lung cancer patients. A study from The Netherlands Cancer Registry found a median time to treatment of 28 days, and a hospital-based registry study conducted in Greece found a median time to treatment of 23 days (7, 17). In regard to Brazil, previous analyses reported a median interval of 35 days between diagnosis and the start of treatment in the private sector and roughly 30 days in public healthcare system (15, 16). The median interval between diagnosis and treatment was almost two-fold greater for individuals diagnosed before the first medical appointment. This difference may be accounted for by the fact that patients who are diagnosed and treated in different health units may suffer delays in the start of treatment, suggesting that an early referral to a specialized center upon lung cancer suspicion may be preferred. Diagnostic procedures (i.e., bronchoscopy, CT-guided biopsy, endobronchial ultrasound) are not always available at primary/secondary institutions. And, notably, pathology reports are often delayed at non-specialized units, as immunohistochemistry is performed offsite, which may lead to delays and misdiagnosis.

Stratifying according to stage, median time to treatment was 33 and 22 days for those without a prior diagnosis in stages III and IV, respectively, and 64 and 54 for those with a prior diagnosis in stages III and IV, respectively. Most of the studies reports data regardless stage at diagnosis and comparisons could not be performed (7, 15, 17). Only Abrao et al. (2017) described that both patients at stages III and IV had, in majority, a delay in the start of treatment shorter than 1.5 months (16). Further analyses are still needed to determine possible differences on time to treatment among patients diagnosed in stages III and IV.

Biomarker analysis is currently recommended for lung cancer (18, 19). Disease guidelines state that molecular testing should be performed as part of the disease diagnosis and progression during targeted therapy (19). The detection of EGFR, BRAF and MET mutations and the analysis of ALK, ROS1, RET, and NTRK translocations have already been incorporated into the diagnostic standards of NSCLC and inhibitors of these kinases are in routine clinical use around the world. In addition to genetic examination, NSCLCs are usually subjected to expression analysis of the programmed death ligand 1 (PD-L1) protein to assess the use of immune checkpoint inhibitors (20). However, available data suggests a lack of this analysis at the start of treatment, which could delay disease management (19).

Unfortunately, access, affordability and incorporation strategies pose significant challenges in low- and middle-income countries. In Brazil, access to molecular testing is limited and data on the occurrence of clinically useful mutations are still scarce, especially in the public healthcare system (3). However, treatment in the private healthcare system is driven by histologic type, molecular findings and PD-L1 expression, in accordance with current recommendations. In Brazil, optimizing strategies to identify the appropriate driver mutations are needed to match the approved molecularly targeted therapies or immunotherapy (9).

Cronemberger et al. (2020) assessed molecular testing and treatment patterns among newly diagnosed patients with locally advanced or metastatic NSCLC across Brazilian cancer centers. The study reported that EGFR mutation testing was performed on 54% of all metastatic adenocarcinoma patients, and 70.4% of those with private health insurance coverage (21). Unfortunately, data on the percentage of patients that had a molecular test ordered was not available in our dataset, or how it could have had an impact on start of treatment delays.

Several variables were assessed to determine associations in regard to the amount of time between the first medical appointment and diagnosis, diagnosis and first treatment, and first treatment and death. The amount of time between the first medical appointment and diagnosis was significantly higher among patients with adenocarcinoma when compared to those with other histological types. In addition, shorter intervals were significantly associated with stage IV (interval between the first medical appointment and diagnosis, diagnosis and first treatment, and first treatment and death). Kourlaba et al. (2019) conducted a study aiming to describe the journey of lung cancer patients in Greece. The author found statistically significant differences for the journeys of patients from the onset of symptoms to the start of treatment and first treatment based on the histological type, however, the impact of the disease stage at diagnosis was not evaluated (7).

Chemotherapy alone was the most common first strategy treatment used among overall (33.8%) and stage IV samples (35.7%). Among stage III group, chemotherapy combined with radiotherapy (concomitant or sequential) was the most frequently used treatment strategy (30.9%), followed by chemotherapy only (28.8%). This data is consistent with a previous analysis conducted in the Brazilian private healthcare system, which shows widespread use of platinum-based regimens (22, 23). Baldotto et al. (2018) analyzed NSCLC patients in the private health sector from 2011 to 2014, and the use of a wide variety of systemic therapies was reported. Among the first-line therapeutic strategies, the most common regimens were bevacizumab + carboplatin + paclitaxel (21.1%), followed by carboplatin + pemetrexed (20.4%) and cisplatin + pemetrexed (17.1%). Radiotherapy was used by 60% of the patients, while surgical procedure was the first-line option in 19.1% of cases and the second-line option in 4.6% (24).

In our analysis, only 0.7% of the patients were using immunotherapy (0.5% among stage III and 0.8% among stage IV). This is a very low estimate since the guidelines of the Brazilian Society of Clinical Oncology recommend this treatment. The first treatment of this class for lung cancer in Brazil was approved in 2017 (25). The use of TKIs could not be assessed as the database only contained intravenous therapy data. Cronemberger et al. (2020) reported that 10.8% of stage IV adenocarcinoma patients tested for EGFR mutations used EGFR-TKIs as a first-line strategy. (20) No study reporting the patterns of immunotherapy use in Brazil have been found to date, thus, further analysis is required to determine these patterns.

Notable strengths and limitations of this study are detailed below. Some of the strengths are related to the analysis of a large database which provides an understanding of the treatment dynamics and journey while pursuing access to disease management. However, the retrospective nature of the study could be related to incomplete records which could impose significant limitations on the analysis. In addition, our analysis did not include the incidence of molecular testing. Until recently, no major therapy improvements had been available to patients with unresectable stage III NSCLC beyond the optimization of chemoradiation regimens. This has now changed with the Brazilian Health Regulatory Agency’s (Anvisa) approval of biological therapies for the treatment of NSCLC patients. The lack of a proper data register in the database may account for the lack of disease management options in our analysis. Our analysis includes stage III/IV patients. However, the treatment plan (curative or palliative) is not clear in the database which adds another limitation on the analysis. Finally, the present analysis does not represent the reality of the entire Brazilian healthcare system.

This study provided an overview of the journey of NSCLC patients in the Brazilian private healthcare setting and revealed a long and variable amount of time until start of treatment. Early patient referral to specialized treatment centers is key to reduce the amount of time before the start of treatment and improve patient outcomes.

Additionally, the results showed that access to innovative treatment strategies such as immunotherapy are still neglected. Further advances in molecular diagnostics and precision oncology are necessary to enable optimal cancer care delivery.

## Data availability statement

Publicly available datasets were analyzed in this study. The database includes hospital data, which is sent voluntarily by public and private establishments and is updated using medical records, public databases and direct contact with individuals. The Integrator Module of the nationwide network of hospital-based cancer registries (RHC) was used. It is coordinated by the Brazilian National Cancer Institute (INCA) and is available to the public online (https://irhc.inca.gov.br/RHCNet/). Annual RHC databases can be extracted in DBF format at: https://irhc.inca.gov.br/RHCNet/visualizaTabNetExterno.action. The databases were opened and stored in Microsoft Excel and analyzed using R (version 4.0.2) software.

## Ethics statement

Ethical approval was not required for the study involving humans in accordance with the local legislation and institutional requirements. Written informed consent to participate in this study was not required from the participants or the participants’ legal guardians/next of kin in accordance with the national legislation and the institutional requirements.

## Author contributions

EC: Conceptualization, Investigation, Supervision, Validation, Writing – review & editing. PB: Conceptualization, Supervision, Validation, Writing – review & editing. MA: Writing – review & editing. MR: Conceptualization, Supervision, Validation, Writing – review & editing. AR: Formal Analysis, Validation, Writing – original draft, Writing – review & editing. FA: Formal Analysis, Writing – original draft, Writing – review & editing. RF: Formal Analysis, Validation, Writing – original draft, Writing – review & editing. AC: Formal Analysis, Writing – review & editing. GS: Formal Analysis, Writing – review & editing.
