# Supplementary material for: The journey of stage III and IV non-small cell lung cancer patients in the Brazilian private healthcare system: a retrospective study
Source: Front Oncol. 2023 Oct 18;13:1257003. doi: 10.3389/fonc.2023.1257003 (PMC10619689; doi:10.3389/fonc.2023.1257003)
Supplement: Supplementary file 1 [file Table_1.docx]

Supplementary Material

**The journey of stage III and IV non-small cell lung cancer patients in the Brazilian private Healthcare System: a retrospective study**

**Erica R. Cerqueira^*^, Paula M. Batista, Milena F. Almeida, Maria A. C. Rego, Ana C. P. Ribeiro-Pereira, Fernando Alencar, Roberta A. Fernandes, Aknar F. C. Calabrich, and Gustavo Schvartsman**

*** Correspondence:** Erica R. Cerqueira
erica.cerqueira@merck.com

# Supplementary Data

## Supplementary Tables

Supplementary Table 1: Full description of the first treatment received at the hospital.

| **Sequence of procedures** | **Overall** | | | | **Stage III** | | | | **Stage IV** | | | |
| --- | --- | --- | --- | --- | --- | --- | --- | --- | --- | --- | --- | --- |
|  | **n** | | **%** | | **n** | | **%** | | **N** | | **%** | |
| Chemotherapy | 3,476 | 33.3 | | 829 | | 28.6 | | 2,647 | | 35.1 | |  |
| None | 1,706 | 16.3 | | 339 | | 11.7 | | 1,367 | | 18.1 | |  |
| Radiotherapy | 1,353 | 13.0 | | 387 | | 13.3 | | 966 | | 12.8 | |  |
| Radiotherapy - Chemotherapy | 1,332 | 12.8 | | 466 | | 16.1 | | 866 | | 11.5 | |  |
| Chemotherapy - Radiotherapy | 857 | 8.2 | | 415 | | 14.3 | | 442 | | 5.9 | |  |
| Surgery - Chemotherapy | 426 | 4.1 | | 129 | | 4.4 | | 297 | | 3.9 | |  |
| Surgery | 333 | 3.2 | | 93 | | 3.2 | | 240 | | 3.2 | |  |
| Surgery - Radiotherapy - Chemotherapy | 185 | 1.8 | | 51 | | 1.8 | | 134 | | 1.8 | |  |
| Surgery - Chemotherapy - Radiotherapy | 122 | 1.2 | | 46 | | 1.6 | | 76 | | 1.0 | |  |
| Other | 102 | 1.0 | | 24 | | 0.8 | | 78 | | 1.0 | |  |
| Surgery - Radiotherapy | 102 | 1.0 | | 21 | | 0.7 | | 81 | | 1.1 | |  |
| Chemotherapy - Other | 42 | 0.4 | | 9 | | 0.3 | | 33 | | 0.4 | |  |
| Chemotherapy - Chemotherapy | 30 | 0.3 | | 4 | | 0.1 | | 26 | | 0.3 | |  |
| Radiotherapy - Chemotherapy - Immunotherapy | 25 | 0.2 | | 5 | | 0.2 | | 20 | | 0.3 | |  |
| Chemotherapy - Surgery | 24 | 0.2 | | 11 | | 0.4 | | 13 | | 0.2 | |  |
| Chemotherapy - Radiotherapy - Chemotherapy | 18 | 0.2 | | 8 | | 0.3 | | 10 | | 0.1 | |  |
| Chemotherapy - Immunotherapy | 17 | 0.2 | | 3 | | 0.1 | | 14 | | 0.2 | |  |
| Radiotherapy - Radiotherapy | 17 | 0.2 | | 3 | | 0.1 | | 14 | | 0.2 | |  |
| Surgery - Other | 16 | 0.2 | | 5 | | 0.2 | | 11 | | 0.1 | |  |
| Radiotherapy - Chemotherapy - Other | 14 | 0.1 | | 4 | | 0.1 | | 10 | | 0.1 | |  |
| Immunotherapy | 13 | 0.1 | | 1 | | 0.0 | | 12 | | 0.2 | |  |
| Radiotherapy - Other | 13 | 0.1 | | 3 | | 0.1 | | 10 | | 0.1 | |  |
| No information | 12 | 0.1 | | 2 | | 0.1 | | 10 | | 0.1 | |  |
| Chemotherapy - Radiotherapy - Surgery | 10 | 0.1 | | 3 | | 0.1 | | 7 | | 0.1 | |  |
| Radiotherapy - Chemotherapy - Radiotherapy | 10 | 0.1 | | 3 | | 0.1 | | 7 | | 0.1 | |  |
| Surgery - Chemotherapy - Other | 10 | 0.1 | | 4 | | 0.1 | | 6 | | 0.1 | |  |
| Surgery - Surgery - Chemotherapy | 10 | 0.1 | | 3 | | 0.1 | | 7 | | 0.1 | |  |
| Chemotherapy - Chemotherapy - Chemotherapy | 9 | 0.1 | | 3 | | 0.1 | | 6 | | 0.1 | |  |
| Surgery - Surgery | 9 | 0.1 | | 1 | | 0.0 | | 8 | | 0.1 | |  |
| Chemotherapy - Chemotherapy - Chemotherapy - Chemotherapy | 9 | 0.1 | | - | | - | | 9 | | 0.1 | |  |
| Chemotherapy - Chemotherapy - Radiotherapy | 7 | 0.1 | | 2 | | 0.1 | | 5 | | 0.1 | |  |
| Chemotherapy - Radiotherapy - Other | 6 | 0.1 | | 2 | | 0.1 | | 4 | | 0.1 | |  |
| Chemotherapy - Radiotherapy - Radiotherapy | 6 | 0.1 | | 2 | | 0.1 | | 4 | | 0.1 | |  |
| Radiotherapy - Chemotherapy - Chemotherapy | 6 | 0.1 | | 1 | | 0.0 | | 5 | | 0.1 | |  |
| Other - Chemotherapy | 6 | 0.1 | | - | | - | | 6 | | 0.1 | |  |
| Radiotherapy - Surgery | 6 | 0.1 | | - | | - | | 6 | | 0.1 | |  |
| Radiotherapy - Immunotherapy | 5 | 0.0 | | 1 | | 0.0 | | 4 | | 0.1 | |  |
| Chemotherapy - Surgery - Radiotherapy | 5 | 0.0 | | - | | - | | 5 | | 0.1 | |  |
| Radiotherapy - Chemotherapy - Surgery | 4 | 0.0 | | 1 | | 0.0 | | 3 | | 0.0 | |  |
| Surgery - Surgery - Radiotherapy - Chemotherapy | 4 | 0.0 | | 2 | | 0.1 | | 2 | | 0.0 | |  |
| Chemotherapy - Surgery - Chemotherapy | 4 | 0.0 | | - | | - | | 4 | | 0.1 | |  |
| Surgery - Chemotherapy - Chemotherapy - Chemotherapy | 3 | 0.0 | | 1 | | 0.0 | | 2 | | 0.0 | |  |
| Other - Radiotherapy | 3 | 0.0 | | - | | - | | 3 | | 0.0 | |  |
| Other - Surgery | 3 | 0.0 | | - | | - | | 3 | | 0.0 | |  |
| Radiotherapy - Surgery - Chemotherapy | 3 | 0.0 | | - | | - | | 3 | | 0.0 | |  |
| Surgery - Radiotherapy - Chemotherapy - Other | 3 | 0.0 | | 3 | | 0.1 | | - | | - | |  |
| Chemotherapy - Chemotherapy - Chemotherapy - Chemotherapy - Chemotherapy - Chemotherapy | 2 | 0.0 | | 1 | | 0.0 | | 1 | | 0.0 | |  |
| Surgery - Chemotherapy - Chemotherapy | 2 | 0.0 | | 1 | | 0.0 | | 1 | | 0.0 | |  |
| Surgery - Chemotherapy - Immunotherapy | 2 | 0.0 | | 1 | | 0.0 | | 1 | | 0.0 | |  |
| Surgery - Radiotherapy - Chemotherapy - Immunotherapy | 2 | 0.0 | | 1 | | 0.0 | | 1 | | 0.0 | |  |
| Chemotherapy - Immunotherapy - Radiotherapy | 2 | 0.0 | | - | | - | | 2 | | 0.0 | |  |
| Chemotherapy - Radiotherapy - Surgery - Other | 2 | 0.0 | | - | | - | | 2 | | 0.0 | |  |
| Immunotherapy - Radiotherapy | 2 | 0.0 | | - | | - | | 2 | | 0.0 | |  |
| Radiotherapy - Chemotherapy - Radiotherapy - Radiotherapy | 2 | 0.0 | | - | | - | | 2 | | 0.0 | |  |
| Surgery - Chemotherapy - Surgery | 2 | 0.0 | | - | | - | | 2 | | 0.0 | |  |
| Surgery - Radiotherapy - Surgery | 2 | 0.0 | | - | | - | | 2 | | 0.0 | |  |
| Surgery - Surgery - Radiotherapy | 2 | 0.0 | | - | | - | | 2 | | 0.0 | |  |
| Surgery - Immunotherapy | 2 | 0.0 | | 2 | | 0.1 | | - | | - | |  |
| Chemotherapy - Chemotherapy - Chemotherapy - Chemotherapy - Chemotherapy | 1 | 0.0 | | - | | - | | 1 | | 0.0 | |  |
| Chemotherapy - Chemotherapy - Chemotherapy - Chemotherapy - Chemotherapy - Chemotherapy - Chemotherapy | 1 | 0.0 | | - | | - | | 1 | | 0.0 | |  |
| Chemotherapy - Chemotherapy - Chemotherapy - Chemotherapy - Chemotherapy - Radiotherapy - Chemotherapy | 1 | 0.0 | | - | | - | | 1 | | 0.0 | |  |
| Chemotherapy - Chemotherapy - Chemotherapy - Radiotherapy | 1 | 0.0 | | - | | - | | 1 | | 0.0 | |  |
| Chemotherapy - Other - Other | 1 | 0.0 | | - | | - | | 1 | | 0.0 | |  |
| Chemotherapy - Other - Radiotherapy | 1 | 0.0 | | - | | - | | 1 | | 0.0 | |  |
| Chemotherapy - Radiotherapy - Chemotherapy - Chemotherapy - Chemotherapy - Chemotherapy - Chemotherapy | 1 | 0.0 | | - | | - | | 1 | | 0.0 | |  |
| Chemotherapy - Radiotherapy - Chemotherapy - Chemotherapy - Chemotherapy - Chemotherapy - Radiotherapy | 1 | 0.0 | | - | | - | | 1 | | 0.0 | |  |
| Chemotherapy - Surgery - Radiotherapy - Surgery | 1 | 0.0 | | - | | - | | 1 | | 0.0 | |  |
| Chemotherapy - Surgery - Surgery | 1 | 0.0 | | - | | - | | 1 | | 0.0 | |  |
| Immunotherapy - Chemotherapy | 1 | 0.0 | | - | | - | | 1 | | 0.0 | |  |
| Other - Chemotherapy - Radiotherapy | 1 | 0.0 | | - | | - | | 1 | | 0.0 | |  |
| Other - Other | 1 | 0.0 | | - | | - | | 1 | | 0.0 | |  |
| Other - Radiotherapy - Chemotherapy | 1 | 0.0 | | - | | - | | 1 | | 0.0 | |  |
| Other - Radiotherapy - Other | 1 | 0.0 | | - | | - | | 1 | | 0.0 | |  |
| Other - Surgery - Chemotherapy | 1 | 0.0 | | - | | - | | 1 | | 0.0 | |  |
| Radiotherapy - Chemotherapy - Surgery - Radiotherapy - Other | 1 | 0.0 | | - | | - | | 1 | | 0.0 | |  |
| Radiotherapy - Other - Chemotherapy | 1 | 0.0 | | - | | - | | 1 | | 0.0 | |  |
| Radiotherapy - Radiotherapy - Radiotherapy | 1 | 0.0 | | - | | - | | 1 | | 0.0 | |  |
| Surgery - Chemotherapy - Chemotherapy - Chemotherapy - Chemotherapy | 1 | 0.0 | | - | | - | | 1 | | 0.0 | |  |
| Surgery - Chemotherapy - Chemotherapy - Radiotherapy | 1 | 0.0 | | - | | - | | 1 | | 0.0 | |  |
| Surgery - Chemotherapy - Chemotherapy - Radiotherapy - Chemotherapy | 1 | 0.0 | | - | | - | | 1 | | 0.0 | |  |
| Surgery - Chemotherapy - Other - Surgery - Other | 1 | 0.0 | | - | | - | | 1 | | 0.0 | |  |
| Surgery - Chemotherapy - Radiotherapy - Chemotherapy | 1 | 0.0 | | - | | - | | 1 | | 0.0 | |  |
| Surgery - Chemotherapy - Radiotherapy - Other | 1 | 0.0 | | - | | - | | 1 | | 0.0 | |  |
| Surgery - Other - Chemotherapy - Immunotherapy | 1 | 0.0 | | - | | - | | 1 | | 0.0 | |  |
| Surgery - Radiotherapy - Chemotherapy - Radiotherapy | 1 | 0.0 | | - | | - | | 1 | | 0.0 | |  |
| Surgery - Radiotherapy - Chemotherapy - Surgery | 1 | 0.0 | | - | | - | | 1 | | 0.0 | |  |
| Surgery - Radiotherapy - Immunotherapy | 1 | 0.0 | | - | | - | | 1 | | 0.0 | |  |
| Surgery - Radiotherapy - Other | 1 | 0.0 | | - | | - | | 1 | | 0.0 | |  |
| Surgery - Radiotherapy - Radiotherapy | 1 | 0.0 | | - | | - | | 1 | | 0.0 | |  |
| Surgery - Surgery - Chemotherapy - Surgery | 1 | 0.0 | | - | | - | | 1 | | 0.0 | |  |
| Surgery - Surgery - Other | 1 | 0.0 | | - | | - | | 1 | | 0.0 | |  |
| Chemotherapy - Radiotherapy - Chemotherapy - Other | 1 | 0.0 | | 1 | | 0.0 | | - | | - | |  |
| Radiotherapy - Chemotherapy - Surgery - Surgery - Radiotherapy - Chemotherapy - Radiotherapy | 1 | 0.0 | | 1 | | 0.0 | | - | | - | |  |
| Surgery - Chemotherapy - Chemotherapy - Surgery - Radiotherapy - Chemotherapy | 1 | 0.0 | | 1 | | 0.0 | | - | | - | |  |
| Surgery - Chemotherapy - Other - Surgery - Chemotherapy | 1 | 0.0 | | 1 | | 0.0 | | - | | - | |  |
| Surgery - Chemotherapy - Surgery - Radiotherapy | 1 | 0.0 | | 1 | | 0.0 | | - | | - | |  |
| Surgery - Radiotherapy - Chemotherapy - Chemotherapy | 1 | 0.0 | | 1 | | 0.0 | | - | | - | |  |
| Surgery - Surgery - Chemotherapy - Radiotherapy | 1 | 0.0 | | 1 | | 0.0 | | - | | - | |  |
